# Supplementary material for: Cx43 carboxyl terminal domain determines AQP4 and Cx30 endfoot organization and blood brain barrier permeability
Source: Sci Rep. 2021 Dec 21;11:24334. doi: 10.1038/s41598-021-03694-x (PMC8692511; doi:10.1038/s41598-021-03694-x)
Supplement: Supplementary file 1 — Supplementary Information. [file 41598_2021_3694_MOESM1_ESM.pdf]

Cx43 carboxyl terminal domain determines AQP4 and Cx30 endfoot organization and blood brain barrier permeability

[1]Antonio Cibelli, [1,2]Randy Stout, [3]Aline Timmermann,
, [1,4]Laura de Menezes, [5]Peng Guo, [6]Karen Maass, [3]Gerald Seifert, [3]Christian Steinhäuser, [1]David C. Spray*,
[7]Eliana Scemes*

Departments of [1]Neuroscience and [5]Anatomy and Structural Biology, Albert Einstein College of Medicine, Bronx, NY, USA.
[2]New York Institute of Technology College of Osteopathic Medicine, Old Westbury NY, USA.
[3]Institute of Cellular Neurosciences, Medical Faculty, University of Bonn, Germany,
[4]Insitute of Biophysics, Federal University of Rio de Janeiro, Brazil.
[5]Cellular Imaging Core Facility, Fred Hutchinson Cancer Research Center, Seattle, Washington, USA.
[6] Department of Cardiology, NYU Medical School, NYC, USA.
[7] Department of Anatomy and Cell Biology, New York Medical College, Valhalla, NY, USA.

SUPPLEMENTARY MATERIAL

ADDITIONAL METHODS.

High Pressure Perfusion (HPP). Six C57Bl6 ($Cx43^{(+/+)}$/$Cx30^{(+/+)}$) mice were perfused with solution containing HRP, as described in Material and Methods. OCT-embedded brains were cryosectioned for HRP staining (using DAB) and bright-field images acquired using a 3D Histech P250 High-Capacity Slide Scanner equipped with a color CMOS camera (VCC-FC60FR19CL, CIS Corp.). Brain section images were evaluated and the density of micro-hemorrhages for each mouse quantified using Case Viewer software.

Immunohistochemistry. Cryosections of HHP brains of $Cx43^{(+/+)}$/$Cx30^{(-/-)}$, $Cx43^{(+/-)}$, $Cx43^{(\Delta/-)}$, and dKO mouse brains were stained for the tight junction protein Claudin-5 using a polyclonal anti-Claudin-5 antibody (1:500; Invitrogen cat# 34-1600). Claudin-5 particle density near brain vessels of mice was determined using super-resolution microscopy (structured illumination microscopy: SIM), as described in Material and Methods. For co-localization of Cx43 and Cx30 at perivascular areas of $Cx43^{(+/+)}$/$Cx30^{(+/+)}$ (WT) mouse brains, cryosections were stained with rabbit anti-Cx43 (1:1000; Sigma, Cat# c6219), mouse anti-Cx30 (1:500; Invitrogen, cat# 33-2500), and goat anti-AQP4 (1:500; Santa Cruz, cat# C19) antibodies. The secondary antibodies employed were Fluor 594 donkey anti-mouse (Invitrogen, cat # R37115), Alexa-Fluor 488 donkey anti-rabbit (Invitrogen, cat# A-21206), and Alexa Fluor 647 donkey anti-goat (Invitrogen, cat # A32849) antibodies.

SUPPLEMENTARY FIGURES

Supplementary Figure S1. **High pressure-induced brain leakage in Cx43$^{(+/+)}$/Cx30$^{(+/+)}$ (WT) mice**. (a) Representative bright-field image showing micro-hemorrhage (HRP leakage revealed with DAB in dark areas: arrow) in brain of WT mouse subjected to high hydrostatic pressure perfusion with a solution containing HRP. (b) Mean ± SEM values of the density of micro-hemorrhages measured from brains of 6 WT mice. No significant difference was detected in terms of density of micro-hemorrhages between WT and Cx43$^{(+/-)}$ (Student's t test, $p = 0.82$). For comparison, values are shown forfor micro-hemorrhage densities measured in Cx43$^{(+/-)}$ brains displayed in Fig. 2b of the main manuscript . Scale bar: 100 µm.

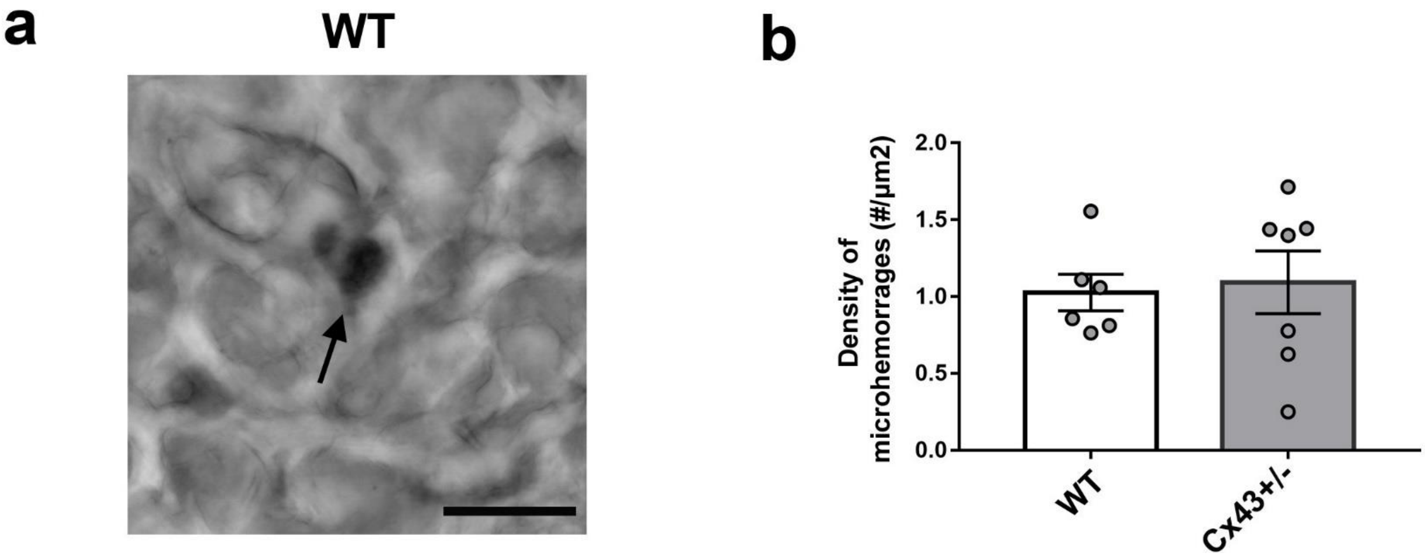

Supplementary Figure S2. Super-resolution microscopy of Cx43 (A-E), AQP4 (F-H), and AQP4ex (I-K) in the astrocyte endfoot in brain sections. (A) Confocal 3D reconstruction from GFAP-YFP mouse (GFAP-Cre; RCE:loxP [RCE:loxP reporter mice (Sousa et al., 2009) were bred with the mGFAP-Cre line (Garcia et al., 2004)] showing cytoplasmic YFP in astrocyte cell body and endfeet (green) and Cx43 immunostaining (red). (B) Zoomed in image of single plane, Cx43 (red) channel only showing lack of sub-500 nm resolution with this confocal microscope and objective/detector. (C) Deconvolved widefield image of a vessel wall at higher magnification than in A. (D) Zoomed image of Cx43 staining from B in widefield with deconvolution (using neighboring z-plane acquisition). (E) SIM reconstruction of the same plaque, note plaque shape and sub-regions can be resolved in E that cannot be resolved in D. (f) Deconvolved single plane widefield image of AQP4 in astrocyte endfeet wrapped around a vessel running diagonally across the image. (g) Inset of the deconvolved immunostained image. (h) Structured Illumination Microscopy reconstruction of the inset region reveals resolution of sub-200 nm features; white arrows indicate portions of the AQP4 signal that can only be recognized with super-resolution imaging parameters. Imaging performed on a Zeiss Elyra S1 with 63X NA1.4 Objective, with 5 grating positions and 40 nm pixels on an EMCCD camera. (i) Confocal 3D reconstruction showing the expression of AQP4ex (green) at the perivascular level in Cx43$^{(+/-)}$ mouse brain sections. Scale bar: 50µm. (j) 3D SIM reconstruction of AQP4ex (green) showing the same vessel of i (white box). Super-resolution imaging allows insights into structural features of the AQP4ex at perivascular level. Scale bar 10 µm. (k) Zoomed 3D SIM image (from j, white box) showing at higher magnification the AQP4ex particles at perivascular level. Scale bar 3 µm.

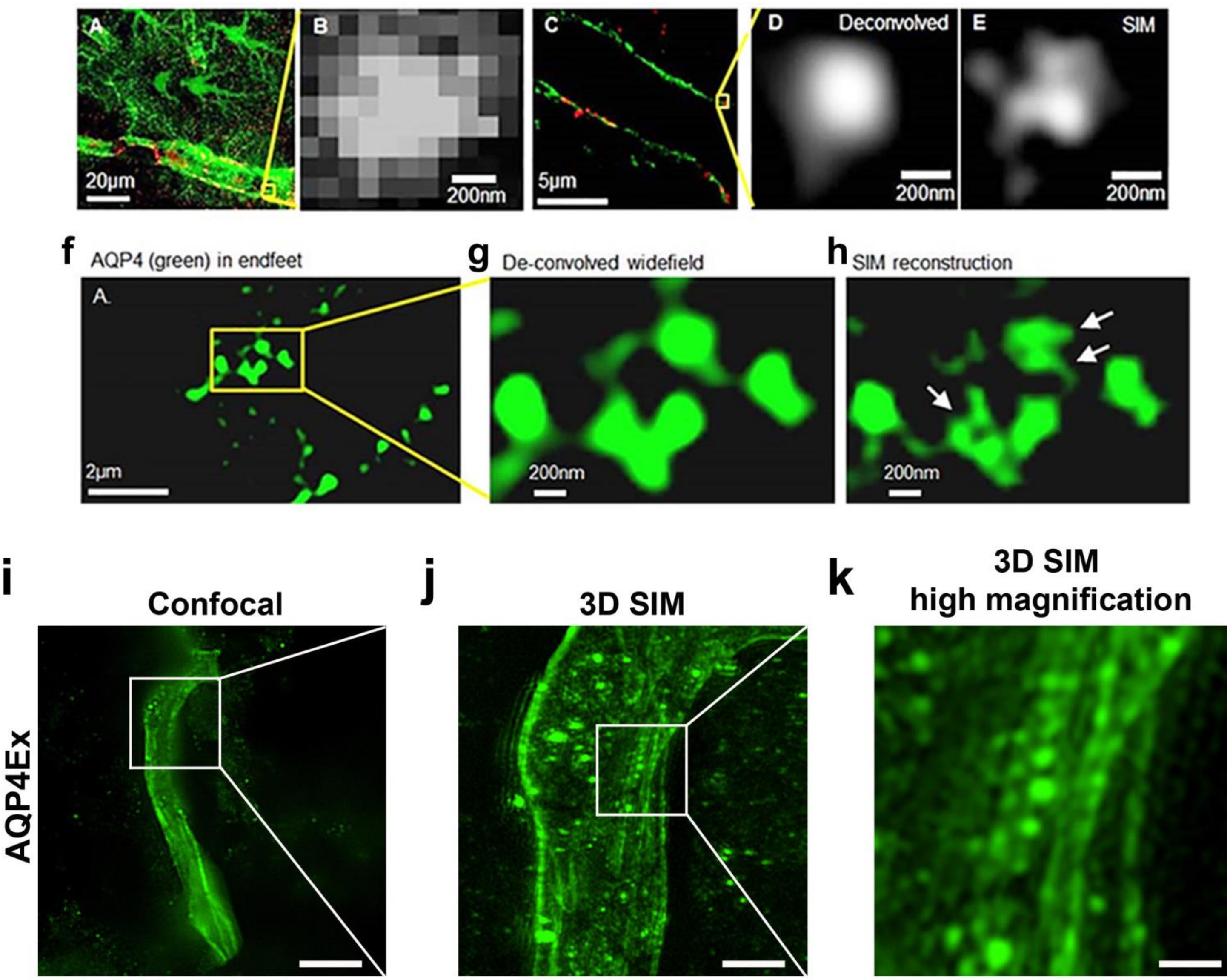

A
20µm
B
200nm
C
5µm
D
Deconvolved
200nm
E
SIM
200nm
f
AQP4 (green) in endfeet
A.
2µm
g
De-convolved widefield
200nm
h
SIM reconstruction
200nm
i
Confocal
AQP4Ex
j
3D SIM
k
3D SIM
high magnification

Supplementary Figure S3. Colocalization of Cx30 and Cx43 in Cx43$^{(+/+)}$/Cx30$^{(+/+)}$ (WT) mouse brains: (a) Representative images showing expression of Cx43 (green) and Cx30 (red) in perivascular areas (blue: AQP4 at astrocyte endfeet) of WT mice. (b) Quantification of the degree of co-localization of Cx43 with Cx30 (m1) and of Cx30 with Cx43 (m2) at perivascular region of WT mice. Note that the degree of overlap of the two connexins with each other was similar to that obtained in Cx43$^{(+/-)}$ mice (see Fig. 8 in main text). Scale bars: 100 µm.

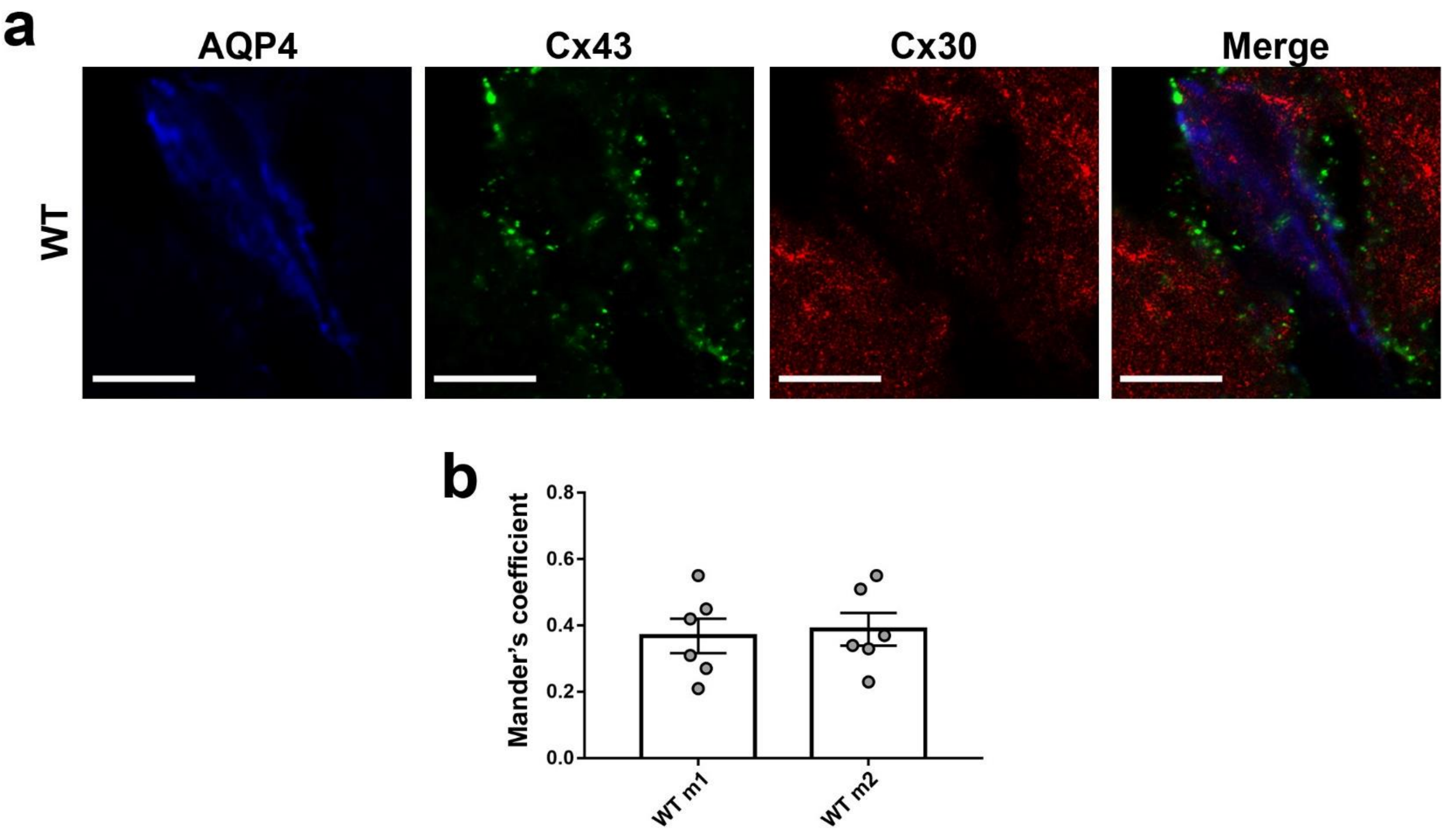

Supplementary Figure S4. Distribution of Claudin-5 in brain sections from Cx43 transgenic mice. (a) 3D SIM reconstruction showing the expression of Claudin-5 (green) and AQP4 (red) in $Cx43^{(+/+)}/Cx30^{(-/-)}$, $Cx43^{(+/-)}$, $Cx43^{(\Delta/-)}$, and dKO brain sections. (b) Mean ± SEM of density of Claudin-5 particles at perivascular areas obtained for each of the transgenic lines (n = 4 mice, 6-10 images and 2000-4000 particles analyzed from each genotype). (c) Mean ± SEM of Claudin-5 particle sizes at perivascular areas measured from in each mouse genotype (n = 4 mice, 6 - 10 images from each genotype). ANOVA followed by Tukey's post hoc test: *p<0.05, **p<0.001. Scale bars: 10 µm.

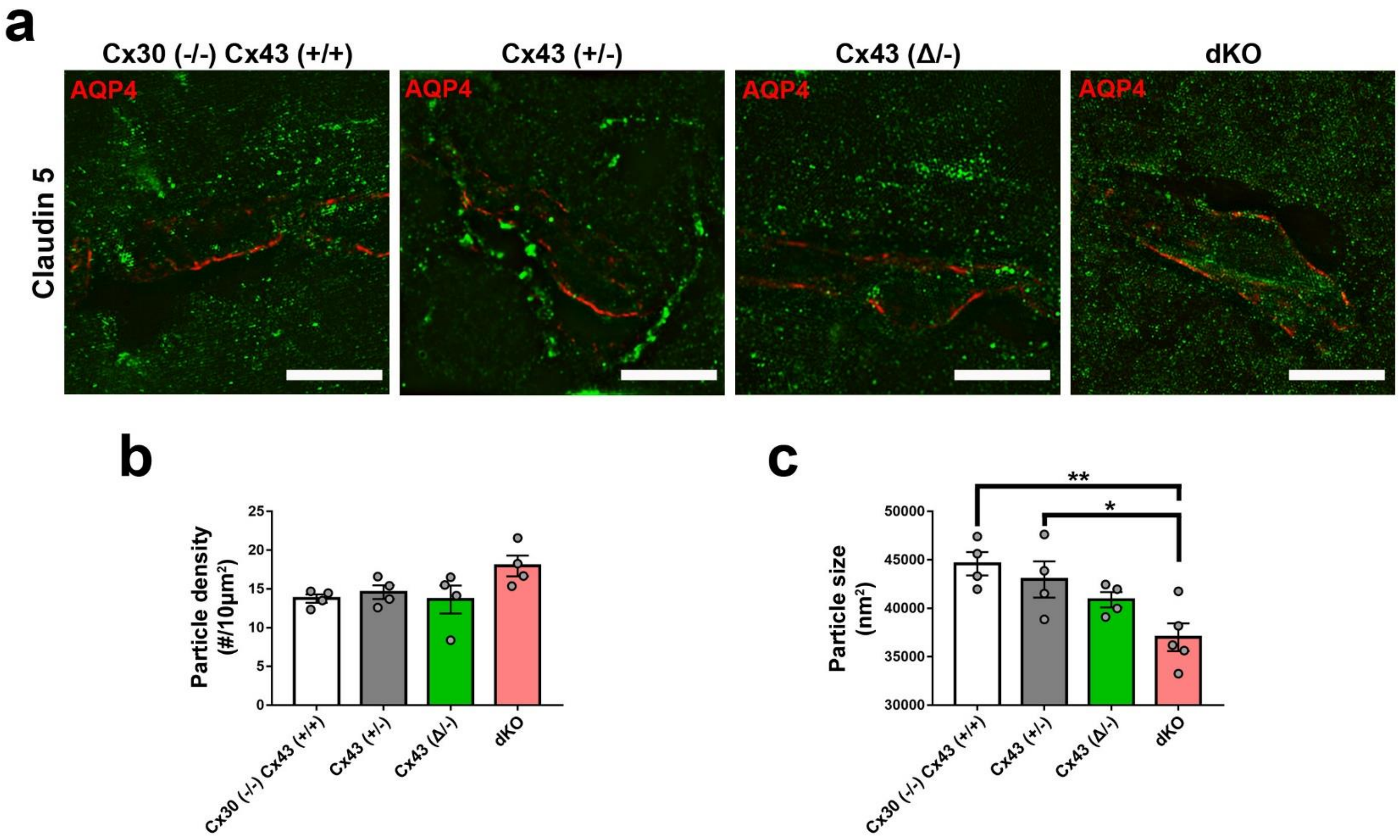

Supplementary Figure S5. Log transformed histograms illustrate normal distribution of sizes of measured particles. Black and red circles represent data from all Cx30$^{(-/-)}$ and dKO brains; slid lines represent best Gaussian fits using GraphPad. (Shapiro–Wilk normality test, ***p < 0.001).

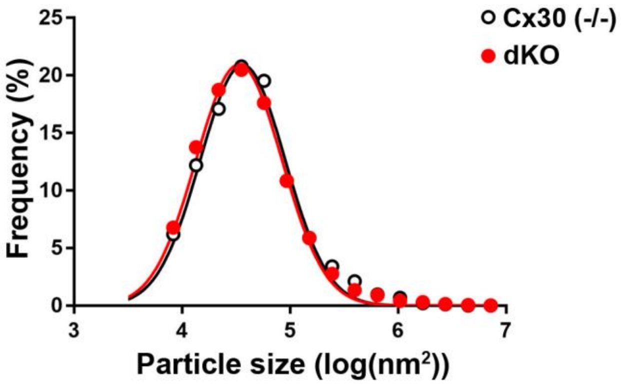

REFERENCES.

Garcia AD, Doan NB, Imura T, Bush TG, Sofroniew MV. 2004. GFAP-expressing progenitors are the principle source of constitutive neurogenesis in adult mouse forebrain. Nat Neurosci 7: 1233–1241.

Sousa VH, Miyoshi G, Hjerling-Leffler J, Karayannis T, Fishell G. 2009. Characterization of Nkx6–2-derived neocortical interneuron lineages. Cereb Cortex 19: 1–I10.
